# Effects of N‐acetyl‐L‐cysteine polysulfides on periodontitis in a mouse model

**DOI:** 10.1002/iid3.959

**Published:** 2023-08-08

**Authors:** Xinxin Sun, Yaru Sun, Sumin Cao, Xueli Liu

**Affiliations:** ^1^ Dental Department, Hejian Hospital of Traditional Chinese Medicine Cangzhou Central Hospital Medical Group Cangzhou Hebei China; ^2^ Dental Clinics Cangzhou Central Hospital Cangzhou Hebei China

**Keywords:** inflammation, NAC‐S2, NF‐κB, periodontitis, TLR4

## Abstract

**Background:**

Polysulfides are reported to be involved in various important biological processes. N‐acetyl‐l‐cysteine polysulfide with 2 sulfane sulfur atoms (NAC‐S2) regulates diverse toll‐like receptor (TLR) signaling pathways. Here, we aimed to determine the role of NAC‐S2 in periodontitis and explore the potential mechanism.

**Methods:**

A periodontitis mouse model was established by ligating the subgingival between the first and second molars in wild‐type, TLR4^‐/‐^, and Myd88^‐/‐^ mice.

**Results:**

NAC‐S2 did not affect the proportion of macrophages (CD11b^+^F4/80^+^) or neutrophils (CD11b^+^GR‐1^+^) in the bone marrow. Mechanically, lipopolysaccharides (LPS), Zymosan A, or poly I: C induced tumor necrosis factor (TNF), interleukin (IL)‐6, and IL‐1β expression in bone marrow‐derived macrophages (BMDMs) could be inhibited by NAC‐S2. On the other hand, NAC‐S2 suppressed the phosphorylation levels of IκB‐α, p65, and IκB kinase (IKK)‐β induced by LPS in BMDMs, while LPS induced phosphorylation of ERK1/2, p38, and transforming growth factor β‐activated kinase 1 (TAK1) could not be affected by NAC‐S2. In wild‐type periodontitis mice, NAC‐S2 administration decreased the cemento‐enamel‐junction–alveolar bone crest (CEJ‐ABC) distance and the relative mRNA expression of TNF, IL‐6, and IL‐1β, while such phenomena could not be observed in TLR4 deficiency or Myd88 deficiency mice.

**Conclusions:**

All of these results indicate that NAC‐S2 ameliorates TLR4/NF‐κB pathway mediated inflammation in mouse periodontitis model.

## Graphical abstract

In established chronic periodontitis mouse model, NAC‐S2 attenuated inflammatory response in TLR4/Myd88‐depedent manner. 2. NAC‐S2 ameliorate inflammation mediated by TLR4/NF‐κB pathway in mouse chronic periodontitis models.

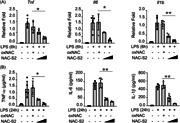

## 1 INTRODUCTION

Periodontitis is developing as one of the most common inflammatory diseases induced by dysbiosis of oral microbiota that affects many people worldwide, and it appears to be a threat to public health.^1^, ^2^ Periodontitis is characterized by the damage or injury of teeth‐supporting structures, such as the periodontal ligament, bone, and gingiva.^3^, ^4^, ^5^ In 2017, the World Workshop on the Classification for Periodontal and Peri‐Implant Diseases and Conditions established a classification system for periodontitis that involved staging and grading.^6^ The main purpose of periodontitis treatment is to eliminate dysbiotic plaque biofilm and reconstruct an appropriate microbiota environment.

Macrophages contribute to periodontal tissue homeostasis, but dysregulated macrophage activation plays a crucial role in periodontitis development.^7^, ^8^ Various studies demonstrate that targeting inflammatory macrophages may serve as a promising strategy for periodontitis treatment.^9^, ^10^, ^11^ From the metabolic levels, lipopolysaccharides (LPS)‐mediated macrophage activation could be inhibited by enhanced intracellular polysulfide level, which is improved by polysulfide donors via presenting the sulfur atoms to acceptor thiols. As an anti‐inflammatory compound, N‐acetylcysteine (NAC) polysulfide can reduce levels of tumor necrosis factor (TNF)‐α, and interleukins (IL‐6 and IL‐1β) by suppressing the activity of nuclear factor kappa B (NF‐κB).^12^

It is worth noting that polysulfide donor treatment, especially NAC polysulfide with 2 sulfane sulfur atoms (NAC‐S2), dramatically suppresses LPS‐mediated inflammation in macrophages.^13^, ^14^ Moreover, NAC‐S2 treatment significantly increases the overall survival of mice with lethal endotoxin shock, suggesting the protecting role of NAC‐S2 against inflammation and lethal endotoxin shock.^15^ However, the effect of NAC‐S2 on periodontitis remains unclear.

In this study, a periodontitis mouse model and bone marrow‐derived macrophages (BMDMs) were utilized to investigate the role of NAC‐S2 in inflammation‐associated periodontitis. Our study demonstrates that NAC‐S2 could suppress the Toll‐like receptor 4 (TLR4)/Myd88‐dependent NF‐κB and IκB kinase (IKK) signaling pathways in periodontitis.

## 2 METHODS

### 2.1 Animals

Wild‐type (WT) C57BL/6 mice and TLR4^‐/‐^ mice on the C57BL/6 background were ordered from GemPharmatech. The Myd88^‐/‐^ mice (Stock NO: 009088) on C57BL/6 background were obtained from Jackson Laboratory. All mice were kept in standard animal facilities with 12/12 h of light/dark cycles and were free to water and diet. The experiments associated with mice were approved by the Ethics Committee of Cangzhou Central Hospital. The age of all mice used in this study was 6–8 weeks. Experimental mice were killed by CO_2_ asphyxiation to achieve euthanasia.

### 2.2 Antibodies and reagents

Anti‐phospho‐NF‐κB p65 (#3033), anti‐phospho‐ERK1/2 (#4370), anti‐ERK1/2 (#4695), anti‐phospho‐p38 (#4511), and anti‐p38 (#8690) antibodies were purchased from Cell Signaling Technology. Anti‐NF‐κB p65 antibody (C‐20, sc‐372) was from Santa Cruz Biotechnology. LPS (*Escherichia coli* 055: B5) was purchased from Sigma‐Aldrich. Polyinosinic‐polycytidylic acid (poly[I: C]) (4287) was ordered from R&D Systems. Zymosan A was purchased from Wako.

### 2.3 NAC‐S2 preparation

**Figure 1:**
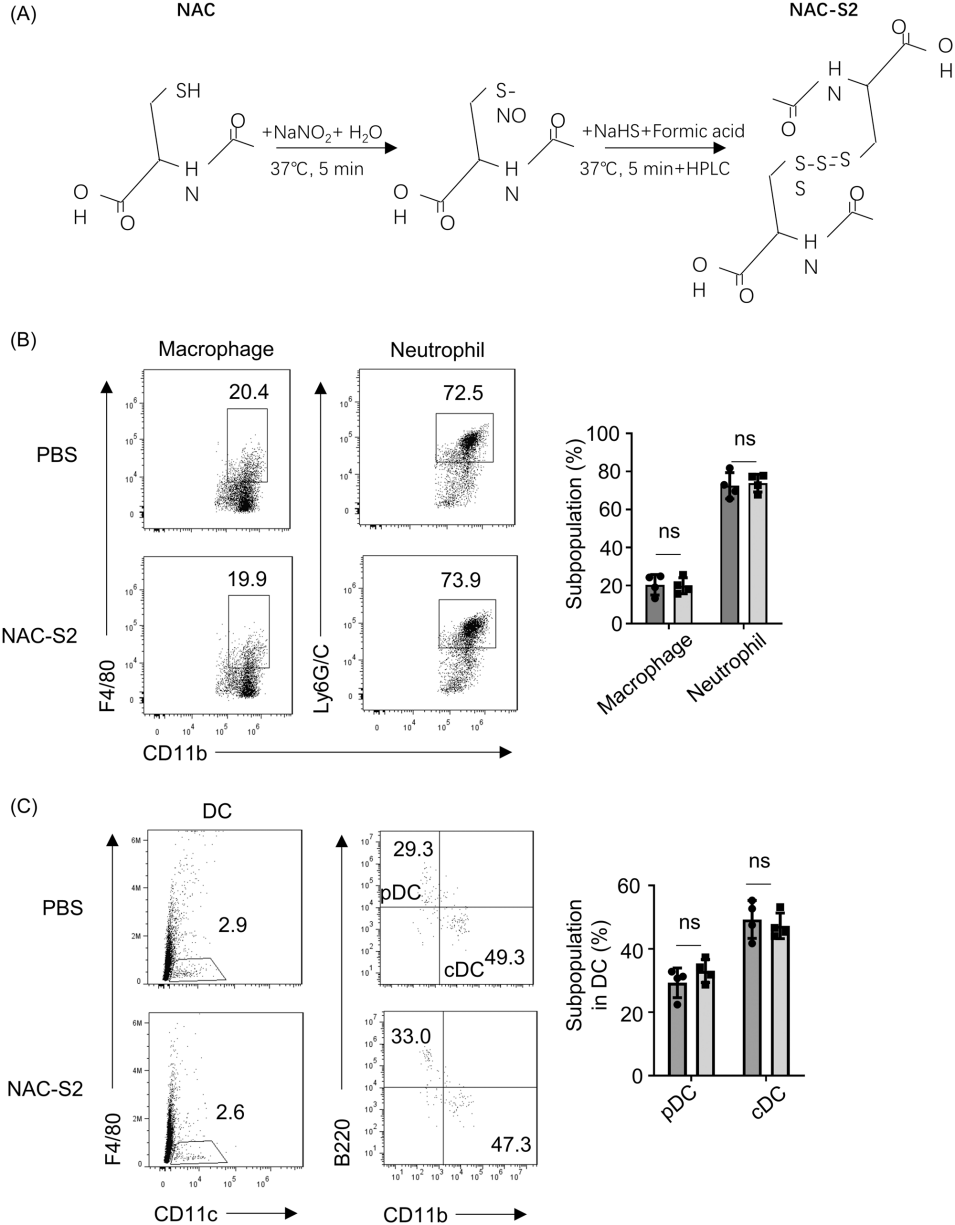
NAC Polysulfides do not affect the development of myeloid cells in the bone marrow. (A) Reaction scheme of the synthesis of NAC polysulfides (NAC‐S2). (B) Flow cytometry analysis of macrophages (CD11b^+^F4/80^+^), and neutrophil (CD11b^+^GR‐1^+^) (*n* = 4). These subpopulations were firstly based on gating the living CD11b^+^ groups. (C) conventional DC (cDC, CD11c^+^CD11b^hi^B220^–^) and plasmacytoid DCs (pDCs, CD11c^+^CD11b^–^B220^+^) in bone marrow from 6 to 8‐weeks mice intraperitoneally injected with PBS (0.1 mL, solvent control) or NAC polysulfides (120 mmol/kg body weight) for 1 week (*n* = 4). These subpopulations were based on gating all living cells first. Data from all panels were presented as representative FACS plots (left) and mean ± SD values based on multiple samples (right). Similar results were obtained in three independent experiments. Two‐tailed Student's *t* tests were performed was applied for statistical comparison between groups. ns, no significance.

NAC‐S2 (Wako) was prepared by reacting NAC with sulfide in the presence of NaNO_2_, following the published protocol.^15^ Briefly, the same concentration of NAC and NaNO_2_ (60 mM, both from Wako) were reacted in H_2_O for 5 min at 37°C, which was further incubated with 45 mM NaHS and 0.2% formic acid for 15 min at 37°C to obtain NAC‐S2 (Figure 1A).

### 2.4 Periodontitis mice model and treatment

Before establishing the periodontitis mouse model, mice were first i.p. injected with phosphate‐buffered saline (PBS) or NAC‐S2 (120 mmol/kg) for 1 week (*n* = 6 in each group). Then the mice were anesthetized using 10% chloralhydrate at the dose of 4 mL/kg. The silk sutures were used to ligate the subgingival between the first and second molars. After the surgery, high sugar in drinking water was supplied to the mice. Twice every week, the ligation was examined, and the ligation would be changed if found loosened. Four weeks after the ligation, the mice were killed for further detection.

### 2.5 CEJ to ABC distance

MILabs U‐CT micro‐CT scan was performed at a 25‐µm voxel size resolution with the following parameters: 360°; 75 ms, exposure; 50 kV, voltage; 0.24 mA, current. Avizo software was utilized for 3‐dimensonally re‐construction. The distance from the cemento‐enamel junction (CEJ) to alveolar bone crest (ABC) was measured in millimeters for the first and second molars of the mandible on both sides.

### 2.6 Bone marrow‐derived macrophages (BMDMs)

Bone marrow cells were first isolated from the femur and tibia of mice, followed by culture in 10% fetal bovine serum (Gibco) and 10 ng/ml macrophage colony‐stimulating factor (M‐CSF) containing RPMI 1640 medium (Life Technology) for 7 days. Later, the differentiated BMDMs were stained with anti‐CD11b and anti‐F4/80 and isolated as CD11b^+^F4/80^+^ cells.

### 2.7 Flow cytometry

Single‐cell suspensions derived from the bone marrow of NAS‐S2 treated mice were subjected to flow cytometry using a FACSAria (BD Bioscience), and the following fluorescence‐labeled antibodies against CD11b (clone M1/70, allophycocyanin‐conjugated, eBioscience), F4/80 (clone Cl: A3‐1, phycoerythrin‐conjugated, eBioscience), and CD11c (clone N418, pacific blue–conjugated, eBioscience). Quadrant gates were set using isotype controls with less than 0.5% background. In the bone marrow, macrophages were defined as CD11b^+^F4/80^+^; neutrophils were defined as CD11b^+^GR‐1^+^; conventional DCs (cDC) were defined as CD11c^+^CD11b^hi^B220^–^ and plasmacytoid DCs (pDCs) were defined as CD11c^+^CD11b^–^B220^+^. All flow cytometry data were analyzed using FlowJo software.

### 2.8 ELISA

The concentrations of TNF (Catalog #: MTA00B, R&D Systems, Inc.), IL‐6 (Catalog #: M6000B, R&D Systems, Inc.), and IL‐1β (Catalog #: MLB00C) in the supernatants of BMDMs were detected by commercial enzyme‐linked immunosorbent assay (ELISA) kits following to manufacturer's protocols. The ELISA kits are purchased from R&D Systems. Each sample was performed as a mean value based on 3‐well repeats. PBS was used as a mock control.

### 2.9 RT‐PCR

RNA was isolated from BMDMs or gingival tissues, followed by reverse transcription‐polymerase chain reaction (RT‐PCR), which was performed by using an SYBR TaqTM kit (Takara). The primers used in the study were shown in Table 1. The $2^{{–\Delta \Delta C}_{t}}$ method was used to detect the relative expression levels of genes. Glyceraldehyde‐3‐phosphate dehydrogenase (GAPDH) expression was used as a control. All sequences of these primers were from the public database of the ORIGENE company.

**Table 1:** The primer sequence used for RT‐PCR.

Primer	Sequences (5′−3′)
TNF	Forward: CAA ATG GCC TCC CTC TCA T
	Reverse: TCC TCC ACT TGG TGG TTT GT
IL‐6	Forward: TGC ACT TGC AGA AAA CAA TC
	Reverse: CCA GTT TGG TAG CAT CCA TC
IL‐1β	Forward: GTA ATG AAA GAC GGC ACA CC
	Reverse: CTT CTT TGG GTA TTG CTT GG
GAPDH	Forward: AAC TGA GGG CTC TGC TCG CT
	Reverse: GTG ACA CAC CGC AAG GCT T

Abbreviation: RT‐PCR, reverse transcription‐polymerase chain reaction.

### 2.10 Western blotting

BMDMs from different groups were lysed in protease inhibitors containing radioimmunoprecipitation assay (RIPA; Thermo Scientific). Protein was separated using 10% gel, and transferred onto nylon membranes. Then, the nylon membranes were blocked using 5% milk, followed by incubating with primary antibodies that were against P‐IκBα, IκBα, P‐p65, p65, P‐ERK1/2, ERK1/2, P‐p38, p38, P‐IKK‐β, IKK‐β, P‐transforming growth factor β‐activated kinase 1 (TAK1), and TAK1 overnight at 4° in the fridge. All antibodies were purchased from Cell Signaling Technology unless specified. Then, the nylon membranes were incubated by HRP‐secondary antibodies. The protein bands were visualized using the chemiluminescence kit (Thermo Scientific).

### 2.11 Statistical analysis

All data were presented as mean ± SD from at least three independent experiments. Statistical analyses represent variations in experimental replicates. Differences between groups were determined by two‐tailed Student's *t* tests, or one‐way ANOVA followed by a Tukey's test. *p* < .05 was believed as statistically significant.

## 3 RESULTS

### 3.1 NAC‐S2 does not influence myeloid cell development in bone marrow

The effect of NAC‐S2 on myeloid cell development was detected in bone marrow by intraperitoneal injecting PBS or NAC‐S2 into wild‐type mice. The percentage of macrophages (CD11b^+^F4/80^+^) or neutrophils (CD11b^+^GR‐1^+^) in bone marrow showed no difference between PBS and NAC‐S2 groups (Figure 1B). In addition, in bone marrow, the proportion of conventional DC (cDC, CD11c^+^CD11b^hi^B220^–^) and plasmacytoid DCs (pDCs, CD11c^+^CD11b^–^B220^+^) between PBS and NAC‐S2 groups did not demonstrate difference (Figure 1C).

### 3.2 NAC‐S2 inhibits various pro‐inflammatory cytokines induced by LPS

**Figure 2:**
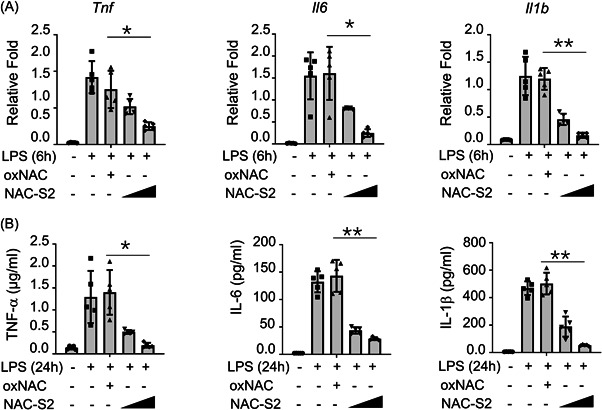
NAC‐S2 inhibited the expression of various pro‐inflammatory cytokines. Primary BMDMs were pretreated with 0.1 or 0.5 mM NAC‐S2 for 24 h, then treated with LPS and/or oxNAC. (A) The expression of indicated cytokines in primary NAC‐S2‐pretreated BMDMs treated with indicated reagents was measured by qRT‐PCR (*n* = 5). (B) ELISA of the indicated cytokines in the supernatants of NAC‐S2‐pretreated BMDMs stimulated with LPS for 24 h (*n* = 5). All data are presented as fold relative to the glyceraldehyde‐3‐phosphate dehydrogenase (GAPDH) mRNA level. Data are presented as mean ± SD values and representative of at least three independent experiments. One‐way ANOVA followed by a Tukey's test was performed for statistical comparison between groups. *p < .05; **p < .01; ***p < .005.

We next deciphered the role of NAC‐S2 in regulating inflammatory cytokines from BMDMs. Oxidized NAC (oxNAC), which lacks sulfane sulfur atoms, was used as the control for NAC‐S2. LPS stimulation led to dramatically increased mRNA expression of TNF, IL‐6, and IL‐1β in BMDMs, while oxNAC administration did not affect the expression of TNF, IL‐6, and IL‐1β. In contrast, NAC‐S2 treatment suppressed LPS‐induced high expression of TNF, IL‐6, and IL‐1β in a dose‐dependent manner (Figure 2A). Consistently, the protein levels of TNF, IL‐6, and IL‐1β in BMDMs were enhanced by LPS stimulation, and were suppressed by NAC‐S2 treatment in a dose‐dependent manner (Figure 2B). These results indicated that NAC‐S2 inhibited various pro‐inflammatory cytokines expression induced by LPS in BMDMs.

### 3.3 NAC‐S2 inhibits various pro‐inflammatory cytokines induced by Zymosan A or poly I:C

**Figure 3:**
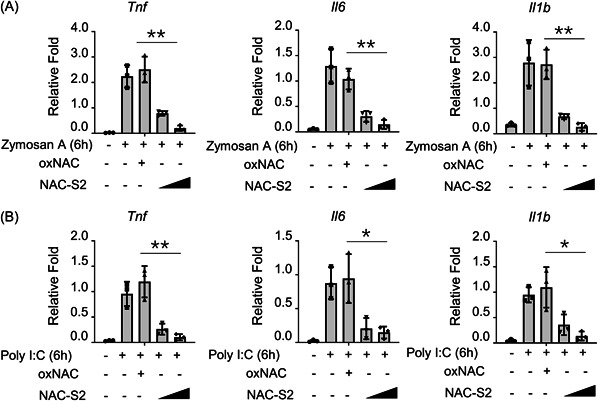
NAC‐S2 also impaired the expression of pro‐inflammatory cytokines induced by various stimulators. Primary BMDMs were pretreated with 0.1 or 0.5 mM NAC‐S2 for 24 h, then treated with zymosan, poly I:C, and/or oxNAC. (A and B) The expression of indicated cytokines in primary NAC‐S2‐pretreated BMDMs treated with indicated reagents was measured by qRT‐PCR (*n* = 3). All data are presented as fold relative to the GAPDH mRNA level. Data are presented as mean ± SD values and representative of at least three independent experiments. One‐way ANOVA followed by a Tukey's test was applied for statistical comparison between groups. *p < .05; **p < .01; ***p < .005. GAPDH, glyceraldehyde‐3‐phosphate dehydrogenase.

LPS‐induced pro‐inflammatory cytokines release is toll‐like receptor 4 (TLR4)‐dependent. We next examined whether NAC‐S2 treatment would influence pro‐inflammatory cytokines release via other TLRs. BMDMs were stimulated with Zymosan A, which activated the TLR2 signaling pathway. As shown in Figure 3A, 6 h after Zymosan A stimulation, the mRNA levels of TNF, IL‐6, and IL‐1β in BMDMs dramatically increased, while NAC‐S2 treatment significantly suppressed these pro‐inflammatory cytokines in a dose‐dependent manner. Similarly, poly(I:C) treatment, which activated TLR2 signaling pathway, enhanced TNF, IL‐6, and IL‐1β expression at mRNA level in BMDMs, while NAC‐S2 treatment significantly inhibited these cytokines in a dose‐dependent manner (Figure 3B). These results indicated that NAC‐S2 inhibits various pro‐inflammatory cytokines induced by Zymosan A or poly I:C.

### 3.4 NAC‐S2 suppresses the phosphorylation of NF‐κB and IKKs signaling

**Figure 4:**
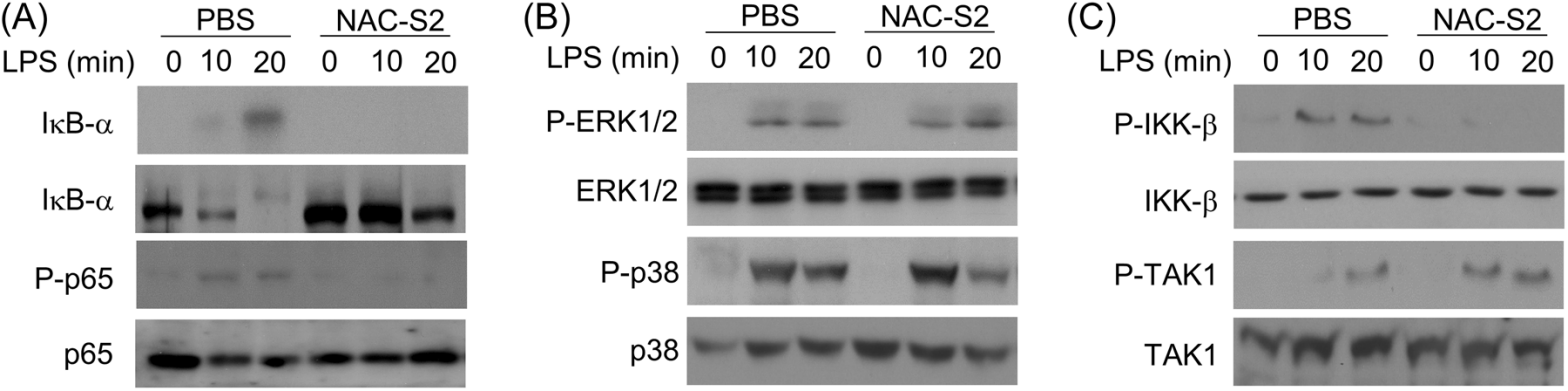
NAC‐S2 suppressed the phosphorylation of IKKs and NF‐kB signal. Primary BMDMs were pretreated with 0.5 mM NAC‐S2 for 24 h, then insulted with LPS. (A and B) Western blot analysis of the indicated phosphorylated (P‐) and total proteins in whole‐cell lysates in NF‐κB signal (A) and MAPKs signal (B) of NAC‐S2‐pretreated BMDMs treated with indicated reagents for the indicated periods. (C) IB analysis of the activated levels of IKKs and TAK1 in total‐cell extracts of indicated BMDMs. BMDMs, bone marrow‐derived macrophages.

We next determined the possible signaling pathways that were involved in NAC‐S2‐mediated inhibition of pro‐inflammatory cytokines release in BMDMs. As shown in Figure 4A, LPS treatment dramatically increased the phosphorylation levels of IκB‐α and p65 in BMDMs, and NAC‐S2 treatment significantly suppressed their phosphorylation levels. LPS treatment also enhanced the phosphorylation levels of ERK1/2 and p38 in BMDMs. However, NAC‐S2 treatment showed no influence on P‐ERK1/2 and P‐p38 levels (Figure 4B).

The phosphorylation level of IKK‐β increased after 10 and 20 min after LPS treatment, while were suppressed by NAC‐S2 treatment (Figure 4C). However, the increased phosphorylation level of TAK1 induced by LPS could not be decreased by NAC‐S2 treatment. These results indicated that NAC‐S2 treatment suppressed NF‐κB and IKKs signal, while did not affect the MAPK signal.

### 3.5 NAC‐S2 attenuates inflammatory response in the periodontitis model

**Figure 5:**
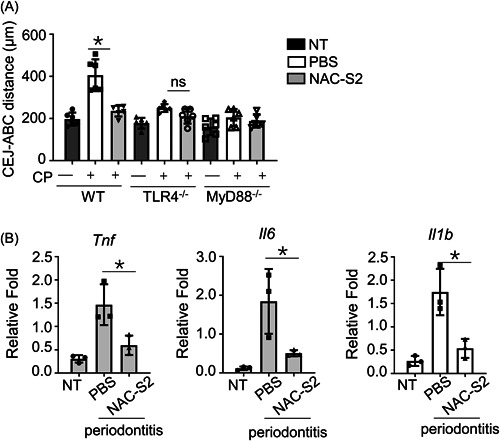
NAC‐S2 ameliorated inflammatory response in the periodontitis model. (A) Periodontitis mice were treated as indicated. 6 to 8‐week mice were intraperitoneally injected with PBS (0.1 mL, solvent control), and NAC polysulfides (120 mmol/kg body weight) for 1 week before inducing the periodontitis model. The cemento‐enamel‐junction–alveolar bone crest was analyzed by micro‐CT (*n* = 6 for each group). Averages and SEM are shown. (B) mRNA levels in gingival tissues from mice with periodontitis were examined by qRT‐PCR. All data are presented as fold relative to the GAPDH mRNA level (*n* = 3). Data are presented as mean ± SD values and representative of at least three independent experiments. One‐way ANOVA followed by a Tukey's test was performed for statistical comparison between groups. *p < .05. GAPDH, glyceraldehyde‐3‐phosphate dehydrogenase.

At last, we determined the effect of NAC‐S2 on periodontitis mouse models that were established in WT mice, TLR4^‐/‐^ mice, and Myd88^‐/‐^ mice. The CEJ‐ABC distance of WT periodontitis mice was much higher than that of normal nontreated (NT) WT mice. NAC‐S2 treatment significantly decreased the CEJ‐ABC distance of WT periodontitis mice. However, in TLR4^‐/‐^ mice and Myd88^‐/‐^ mice, NAC‐S2 treatment could not decrease CEJ‐ABC distance anymore. All of these indicated the effect of NAC‐S2 on periodontitis was TLR4/Myd88 dependent (Figure 5A).

Moreover, the effect of NAC‐S2 on pro‐inflammatory cytokines of gingival tissues from periodontitis was further detected. As expected, the mRNA levels of TNF, IL‐6, and IL‐1β dramatically increased in periodontitis mice as compared with control NT mice, while NAC‐S2 treatment significantly suppressed their expression levels in periodontitis mice (Figure 5B).

## 4 DISCUSSION

In this study, the effect of NAC polysulfides on inflammation‐mediated periodontitis is explored. Our results indicate that NAC‐S2 does not influence myeloid cell development in the bone marrow of WT mice. While in the established periodontitis mouse model, NAC‐S2 administration significantly decreases CEJ to ABC distance and suppresses the expression levels of pro‐inflammatory cytokines including TNF, IL‐6, and IL‐1β. In BMDMs, NAC‐S2 administration suppresses LPS, Zymosan A, or poly I:C‐induced higher expression of pro‐inflammatory cytokines. Mechanically, our investigation demonstrates that NAC‐S2 could suppress the phosphorylation of IKKs and NF‐κB signals.

Inflammation is widely believed to be associated with the development of periodontitis.^16^, ^17^ In patients with severe periodontitis, the serum concentrations of pro‐inflammatory cytokines such as IFN‐γ, IL‐2, TNF‐α, and IL‐1β are much higher than that of healthy controls, which is also observed in gingival tissue biopsies.^18^ In addition, the IL‐1β/IL‐10 ratio and TNF‐α/IL‐4 ratio in the biopsies of periodontitis patients are strongly associated with the severity of periodontitis.^19^ Similarly, in our study, we find that NAC‐S2 treatment significantly suppresses the mRNA expression levels of TNF, IL6, and IL‐1β in gingival tissues of periodontitis mice, indicating the protecting role of NAC‐S2 in periodontitis.

TLR family members are crucial in identifying infiltrating microbiota and in reprogramming innate immunity.^20^, ^21^ LPS is the main component of the Gram‐negative bacteria cell wall, which could be recognized by TLR4 expressed on various cell types to activate inflammatory cytokine expression.^22^, ^23^ The signaling transduction of LPS‐TLR4 is mediated through the Myd88 pathway, and this transduction finally results in inflammatory cytokine expression.^24^, ^25^ Zhang and colleagues report that in LPS‐stimulated RAW264.7 cells, NAC‐S2 treatment significantly suppresses TNF‐α and IFN‐γ production. When detecting the signaling pathways, they find that NAC‐S2 treatment markedly suppresses the protein expression of IKK/NF‐κB axis, while NAC‐S2 treatment significantly enhances the protein levels of JNK, ERK, and MAPK signaling pathways.^15^ IKK plays critical roles in diseases.^26^ Consistently, our study shows that NAC‐S2 treatment significantly suppressed the LPS‐induced higher phosphorylation levels of IκBα and p65 expression in BMDMs. However, in our study, we find no influence of NAC‐S2 treatment on P‐ERK1/2 and P‐p38 levels, which indicates that MAPK signal pathway is not affected by NAC‐S2 in BMDMs.

The role of NAC‐S2 on periodontitis is TLR4/Myd88 dependent. When using TLR4^‐/‐^ or Myd88^‐/‐^ mice to establish the periodontitis model, we find that CEJ to ABC distance is much lower than that of WT mice. More importantly, NAC‐S2 treatment shows no influence on CEJ to ABC distance in TLR4^‐/‐^ or Myd88^‐/‐^ mice periodontitis model. Consistently, the previous study reports that LPS‐induced inflammation in macrophages is attenuated by polysulfide donor treatment via suppressing the TLR4 signaling pathway.^15^, ^27^

It is important to note the limitation of this study. First, the effect of NAC‐S2 on periodontitis is achieved in a TLR4/Myd88‐dependent manner. This is only evidenced by the CEJ to ABC distance. However, the effect of NAC‐S2 on pro‐inflammatory cytokines in TLR4^‐/‐^ or Myd88^‐/‐^ mice is not detected here. Second, a clinical study is needed to verify the results from mouse models. Third, it would be interesting to explore the effects of NAC‐S2 treatment in different ages of mice to see if there is relationship between age and the activities of NAC‐S2.

In summary, this study demonstrates that NAC‐S2 inhibits various pro‐inflammatory cytokines and periodontitis in mouse model in TLR4/Myd88‐dependent manner. Mechanically, NAC‐S2 suppresses the phosphorylation of IKKs and NF‐κB signal. This study supplies experimental evidence for the potential usage of NAC‐S2 in periodontitis.

## 5 CONCLUSION

NAC‐S2 ameliorate inflammation mediated by TLR4/NF‐κB pathway in mouse periodontitis models.

## AUTHOR CONTRIBUTIONS

*Data curation*: Xinxin Sun, Yaru Sun, Sumin Cao, and Xueli Liu. *Analysis*: Xinxin Sun, Yaru Sun, Sumin Cao, and Xueli Liu. *Drafting of the manuscript*: Xinxin Sun. *Concept, design of the study*: Xinxin Sun. All authors approved the publication of the manuscript.

## CONFLICT OF INTEREST STATEMENT

The authors declare no conflict of interest.

## ETHICS STATEMENT

The experiments associated with mice were approved by the ethics committee of Cangzhou Central Hospital. This study was performed in strict accordance with the NIH guidelines for the care and use of laboratory animals (NIH Publication No. 85‐23 Rev. 1985).

## Supporting information

Supporting information.

## Data Availability

The raw data supporting the conclusions of this article will be made available by the authors without undue reservation. The data sets generated during and/or analyzed during the current study are available from the corresponding author upon reasonable request.
